# Ketoreductase Catalyzed (Dynamic) Kinetic Resolution for Biomanufacturing of Chiral Chemicals

**DOI:** 10.3389/fbioe.2022.929784

**Published:** 2022-06-30

**Authors:** Chenming Huang, Junling Liu, Jiali Fang, Xian Jia, Zhendong Zheng, Song You, Bin Qin

**Affiliations:** ^1^ Wuya College of Innovation, Shenyang Pharmaceutical University, Shenyang, China; ^2^ Department of Oncology, General Hospital of Northern Theater Command, Shenyang, China; ^3^ School of Pharmaceutical Engineering, Shenyang Pharmaceutical University, Shenyang, China; ^4^ School of Life Sciences and Biopharmaceutical Sciences, Shenyang Pharmaceutical University, Shenyang, China

**Keywords:** ketoreductase, biocatalysis, asymmetric reaction, kinetic resolution, dynamic kinetic resolution

## Abstract

Biocatalyzed asymmetric reduction of ketones is an environmentally friendly approach and one of the most cost-effective routes for producing chiral alcohols. In comparison with the well-studied reduction of prochiral ketones to generate chiral alcohols with one chiral center, resolution of racemates by ketoreductases (KREDs) to produce chiral compounds with at least two chiral centers is also an important strategy in asymmetric synthesis. The development of protein engineering and the combination with chemo-catalysts further enhanced the application of KREDs in the efficient production of chiral alcohols with high stereoselectivity. This review discusses the advances in the research area of KRED catalyzed asymmetric synthesis for biomanufacturing of chiral chemicals with at least two chiral centers through the kinetic resolution (KR) approach and the dynamic kinetic resolution (DKR) approach.

## 1 Introduction

**FIGURE 1:**
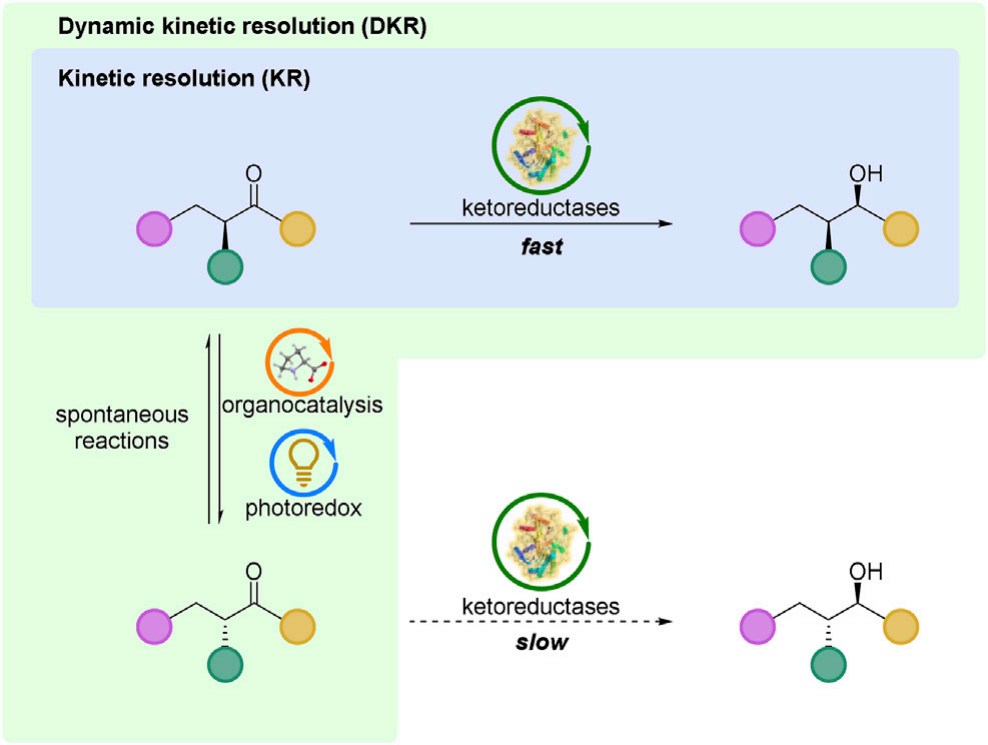
Kinetic resolution (KR) and dynamic kinetic resolution (DKR) based on ketoreductases catalyzed reductions. For both KR and DKR, the racemic substrate was used as the start material, and only one of the enantiomers with a faster rate was highlighted in KR process for a clear explanation.

Since the discovery of microbial resolution of tartaric acid in 1858 by Louis Pasteur (Pasteur, 1858), the kinetic resolution (KR) of stereoisomers has become as one of the most important methods to obtain optical pure compounds, especially in the pharmaceutical industry due to the regulatory requirements for single stereoisomer of chiral drugs. In the kinetic resolution, the enantiomers of a racemic substrate react by chiral catalysts or enzymes at different rates; one of the enantiomers with a much faster rate completes the reaction first while the other enantiomer with a slower reaction rate is left (Figure 1). In theory, the maximum yield of the formed product is 50% (Keith et al., 2001). There are some common kinetic resolution reactions, such as 1) acylative/deacylative resolution (Sheldon, 1996; Somfai, 1997); 2) oxidative kinetic resolution (Komatsu et al., 1992; Hashiguchi et al., 1995); 3) reductive kinetic resolution (Harada et al., 1990; Kurosu et al., 1998); 4) organometallic kinetic resolution (Hayashi et al., 1986; Morken et al., 1994); 5) kinetic resolution of epoxides (Martin et al., 1981; Hodgson et al., 1996).

Owing to the limitation of a maximum 50% theoretical yield, it is urgently needed to increase the yields of KR methods. The first clear dynamic kinetic resolution (DKR) was achieved by Weygand and co-workers in 1966, in which the synthesized dipeptides from racemic azlactones were unequal (Weygand et al., 1966). As shown in Figure 1, DKR also relies on enantiomers of a racemic substrate that react (by catalysts) at different rates, but the great difference of DKR is the rapidly dynamic equilibrium (occurred spontaneously, or by catalysis) between the enantiomers of a racemic substrate. Under this circumstance, the certain production of the resolution reaction can achieve 100% theoretically.

Ketoreductases catalyzed reductions of ketones into chiral alcohols are the widely used and important biocatalytic reactions (Kaluzna et al., 2005a; Brenna, 2013; Hollmann et al., 2021; Li et al., 2021; Musa, 2022). The increasing studies on protein engineering or directed evolution (the Nobel prize in chemistry 2018) (Arnold, 2019) of KREDs, and combining KREDs with other catalysts, such as biocatalysts, metal catalysts (the Nobel prize in chemistry 2001) (Noyori, 2002), organocatalysts (the Nobel prize in chemistry 2021) (Hargittai, 2022), and photocatalysts (DeHovitz et al., 2020), further encourage the application of KREDs in asymmetric synthesis. For instance, several blockbuster drugs, such as atorvastatin, montelukast, and crizotinib, could be synthesized by the KREDs catalyzed process in the pharmaceutical industry (Bornscheuer et al., 2012; Devine et al., 2018; Campos et al., 2019). In comparison with the well-known reduction of prochiral ketones to chiral alcohols with one chiral center, (dynamic) kinetic resolution of racemates by KREDs to produce chiral compounds with at least two chiral centers is also an attractive approach in asymmetric synthesis (Stewart, 2001; Kalaitzakis and Smonou, 2013; Applegate and Berkowitz, 2015; Yang et al., 2022). In this review, we describe the advances in the research area of KREDs catalyzed asymmetric synthesis of chiral compounds with at least two stereocenters through KR and DKR approaches. Of the DKR, the dynamic equilibrium between the enantiomers of substrates could be archived by spontaneous reaction, organocatalysis, and photoredox reactions. For a clearer overview, the ketoreductases catalyzed oxidative KR of chiral alcohols, and the desymmetrization of achiral materials (Chen et al., 2019; Li J. et al., 2020) were not involved in this review.

## 2 Ketoreductase Catalyzed Kinetic Resolution

**FIGURE 2:**
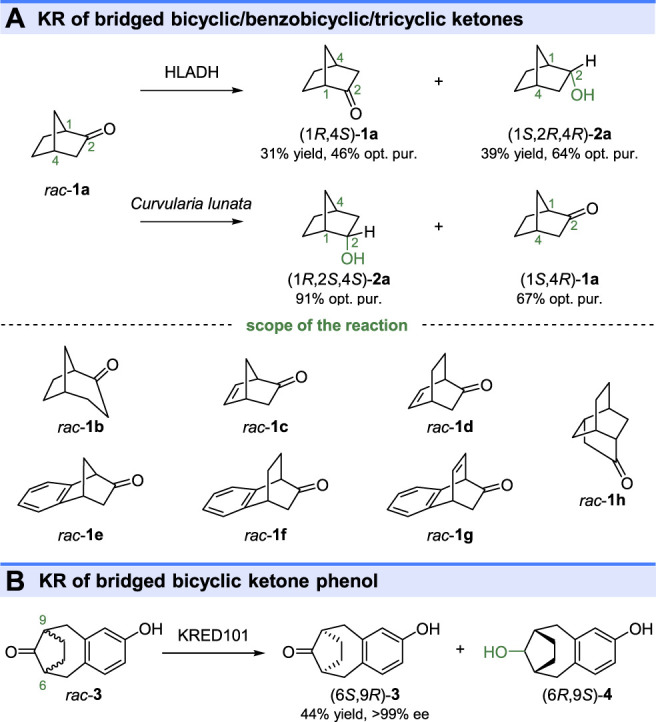
Kinetic resolution of **(A)** bridged bicyclic/benzobicyclic/tricyclic ketones and **(B)** bridged bicyclic ketone phenol.

An early study of KREDs catalyzed kinetic resolution was reported by Jones and Irwin (1976), in which, the efficient KR of bicyclic ketones *rac*-2-norbornanone (*rac*-**1a**, in Figure 2) and *rac*-exo-norbornanol (*rac*-**1b**, Figure 2) were carried out by horse liver alcohol dehydrogenase (HLADH) (Irwin and Jones, 1976) (Figure 2). Similarly, the KR of bicyclic ketones *rac*-**1a** could also be performed by *Curvularia lunata* and *Rhodotorula rubra* (Nakazaki et al., 1980)*.* Interestingly*,* the *Curvularia lunata* catalyzed KR reaction displayed opposite stereoselectivity toward *rac*-**1a** in comparison with the reaction by HLADH, yielding (1*R*,2*S*,4*S*)-**2a** with a 91% optical purity (ee value was not described in the study) as the reductive product. The microbial reduction and HLADH catalyzed reduction could also resolve several other ketones, such as bicyclic ketones *rac*-**1b-d**, benzobicyclic ketones **1e-g,** and tricyclic ketone **1h**. As shown in Figure 2B, a pharmaceutically important keto-phenol (6*S*,9*R*)-**3** could also be obtained by KRED 101 catalyzed KR of bridged bicyclic ketone phenol *rac*-**3** at a 1 kg scale (Truppo et al., 2006).

**FIGURE 3:**
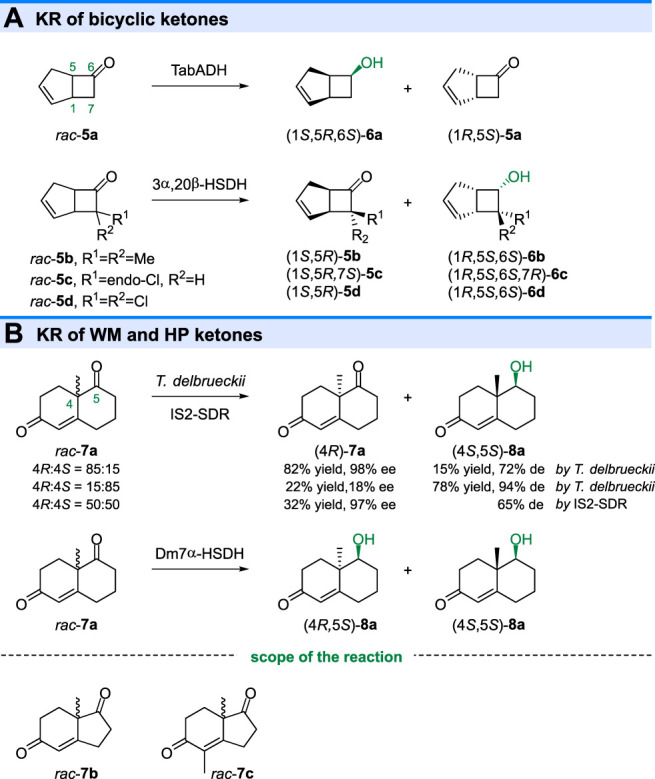
Kinetic resolution of **(A)** bicyclic ketones and **(B)** WM/HP ketones.

In 1985 and 1986, the KR of bicyclic ketones with [3.2.0]-ring system were also reported (Butt et al., 1985; Davies et al., 1986). As shown in Figure 3A, reduction of the *rac*-**5a** with an alcohol dehydrogenase from *Thermoanaerobium brockii* (TabADH) gave alcohol (1*S*,5*R*,6*S*)-**6a** in high optical purity (>95% ee) (Butt et al., 1985). However, another enzyme 3α,20β-hydroxysteroid dehydrogenase (3α,20β-HSDH) from *Streptomyces hydrogenans* displayed opposite selectivity toward 7-substituted bicyclo[3.2.0]heptenones (**5b-d**), giving (6*S*)-alcohols and leaving unreacted (1*S*,5*R*)-ketones (Davies et al., 1986), while (1*R*,5*S*)-ketone was unreacted in TabADH catalyzed KR of *rac*-**5a**.

The bicyclic Wieland-Miescher ketone (WMK, **7a**) and Hajos-Parrish ketone (HPK, **7b**) are important building blocks in the synthesis of natural products, including terpenoids and steroids. Ketoreductase catalyzed KR of racemic WMK or HPK is an efficient strategy to prepare enantiomerically enriched ketones. Sugai and coworkers reported that the *Torulaspora delbrueckii* IFO 10921 could only reduce the carbonyl group of the (4*S*)-**7a**, giving unreacted (4*R*)-**7a** (98% ee) and reduced (4*S*,5*S*)-**8a** (94% de) when (4*R*)-**7a** (70% ee) and (4*S*)-**7a** (70% ee) were used, respectively (Fuhshuku et al., 2000). The KR of *rac*-**7a** (4*R*:4*S* = 50:50) could also be achieved by IS2-SDR (from Icelandic metagenome) catalyzed reaction (Bertuletti et al., 2021). Although *T. delbrueckii* and IS2-SDR displayed the same diastereo preference toward (4*R*)-**7a** and (4*S*)-**7a** in KR reaction, another enzyme Dm7α-HSDH (from *Deinococcus marmoris*) (Bertuletti et al., 2021) could completely convert *rac*-**7a** into two diastereoisomers (4*S*,5*S*)-**8a** and (4*R*,5*S*)-**8a,** indicating the low diastereoselectivity. The KR of HPK (*rac*-**7b**) and its derivative (*rac*-**7c**) have also been reported by using microbial reduction and ketoreductases catalyzed reduction (Hioki et al., 2000; Janeczko et al., 2010).

**FIGURE 4:**
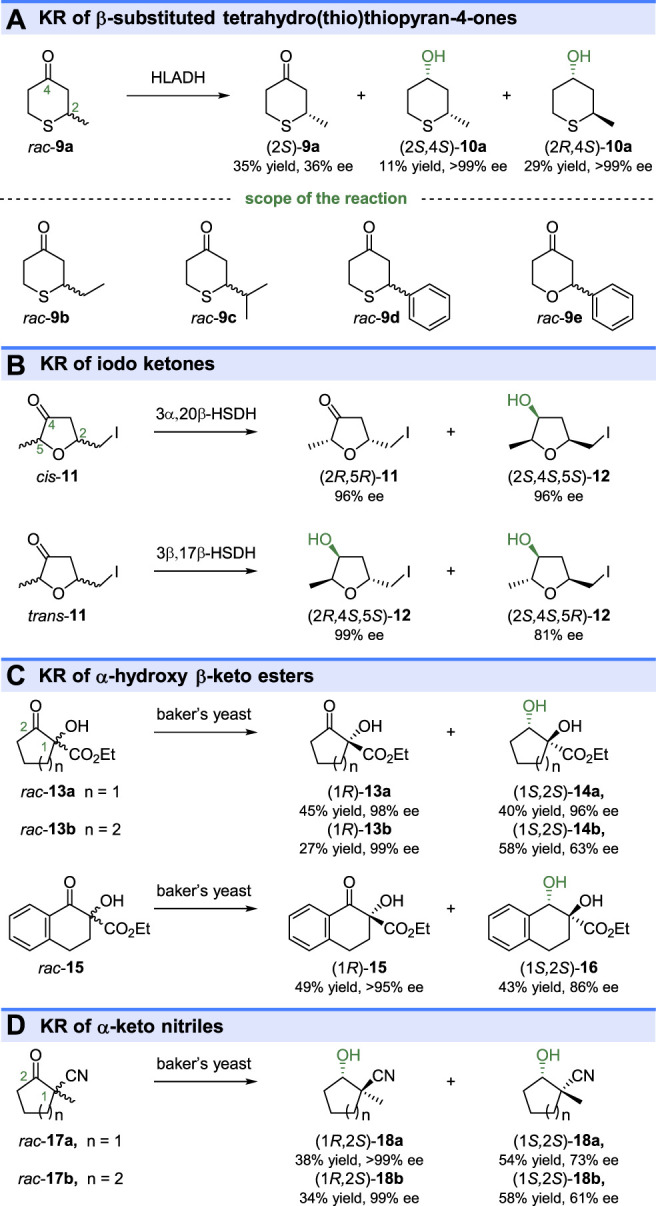
Kinetic resolution of substituted monocyclic ketones. **(A)** KR of β-substituted tetrahydro(thio)thiopyran-4-ones, **(B)** KR of iodo ketones, **(C)** KR of α-hydroxy β-keto esters, and **(D)** KR of α-keto nitriles.

The substituted monocyclic ketones could also be resolved by ketoreductases catalyzed reduction. As shown in Figure 4A, HLADH showed the ability to KR of β-substituted tetrahydrothiopyran-4-ones (*rac*-**9a-d**) (Davies and Jones, 1979) and 2-phenyltetrahydropyran-4-one (*rac*-**9e**) (Sotolongo et al., 1999). Although both (2*S*)-**9a** and (2*R*)-**9a** could be reduced by HLADH, the resultant (2*S*,4*S*)-**10a** and (2*R*,4*S*)-**10a** were optical pure, indicating the high stereo preference of HLADH to yield (4*S*)-alcohols. In 1991, chemoenzymatic synthesis of the stereoisomeric muscarines, one of the widely studied alkaloids, was reported based on KREDs catalyzed kinetic resolution (De Amici et al., 1991). Reduction of iodo ketones *cis*-**11** by using 3α,20β-hydroxysteroid dehydrogenase (3α,20β-HSDH) from *Streptomyces hydrogenans* gave the reduced iodo alcohol (2*S*,4*S*,5*S*)-**12** (96% ee) and unreacted ketone (2*R*,5*R*)-**11** (Figure 4B). The other stereoisomers, iodo alcohols (2*R*,4*S*,5*S*)-**12** (>99% ee) and (2*S*,4*S*,5*R*)-**12** (81% ee) could be obtained by 3β,17β-hydroxysteroid dehydrogenase [(3β,17β)-HSDH, from *Pseudomonas testosterone*] catalyzed KR of *trans*-**11** (Figure 4B). The monocyclic α-alkyl-α-hydroxy-β-keto esters (*rac*-**13a-b** and **15**) could also be effectively resolved through baker’s yeast catalyzed reduction (Buisson et al., 2000) (Figure 4C). Another similar compound, 1-methyl-2-oxocycloalkane carbonitrile (*rac*-**17a-b**) have been reduced by the fungus *Mortierella isabellina* NRRL 1757 through a parallel kinetic-resolution process (Dehli and Gotor, 2002) (Figure 4D). The parallel kinetic-resolution was also found in the ketoreductase catalyzed reduction of β-hydroxyketones (Baer et al., 2009; Sonoike et al., 2012).

**FIGURE 5:**
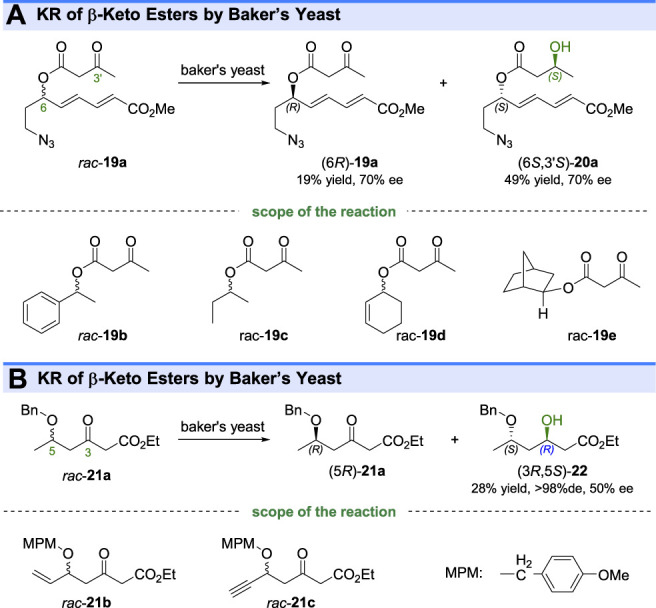
Kinetic resolution of β-keto esters **(A,B)**.

In addition to the kinetic resolution of the aforementioned cyclic ketones, some studies about the KR of aliphatic ketones by ketoreductases have also been reported. For example, Murry and coworkers reported that the baker’s yeast-mediated reduction could resolve the β-keto esters **19a-e** by conversion of (6*S*)-ketones to corresponding β-hydroxy esters (6*S*,3′*S*)-**20a-e** (Hudlicky et al., 1991) (Figure 5A). Interestingly, the selective reduction indicated that the microbial reaction could recognize the remote chiral centers beyond the reactive groups. Similar KR reactions were done by other groups, who also used baker’s yeast for kinetic resolution of β-keto esters (Eh and Kalesse, 1996) (Figure 5B).

## 3 Ketoreductases Involved in Dynamic Kinetic Resolution Processes

For kinetic resolution, one of the most unfavorable disadvantages is the yield limitation of <50%. In addition to that, the low selectivity of ketoreductases might also produce the undesired stereoisomers. In addition, the need for the separation of resultant products and unreacted substrates is also an issue. To overcome the disadvantages in KR, especially the yield limitation, one of the most important strategies that have been developed is dynamic kinetic resolution. In recent decades, ketoreductases based on DKR of racemic substrates to obtain products with two chiral centers have appeared a lot. Racemic functional compounds such as ketoesters, ketoamines, ketothioesters, keto arylphosphonates, and cyclic ketones could be efficiently resolved with excellent yields and stereoselectivities.

As mentioned in the introduction section, the DKR relies on the rapid interconversion (occurring spontaneously or by catalysis) of substrate enantiomers. For racemic ketones capable of spontaneous racemization or base-catalyzed racemization, their common feature is that there is an acidic proton at the α-position of ketones. The stereochemistry can often be established on the adjacent carbon, due to keto-enol tautomerism and selective reduction one of the ketone enantiomers by enzymes (Hanson et al., 2016).

### 3.1 Ketoreductases Involved in DKR of α-Heteroatom-Substituted β-Keto Esters

**FIGURE 6:**
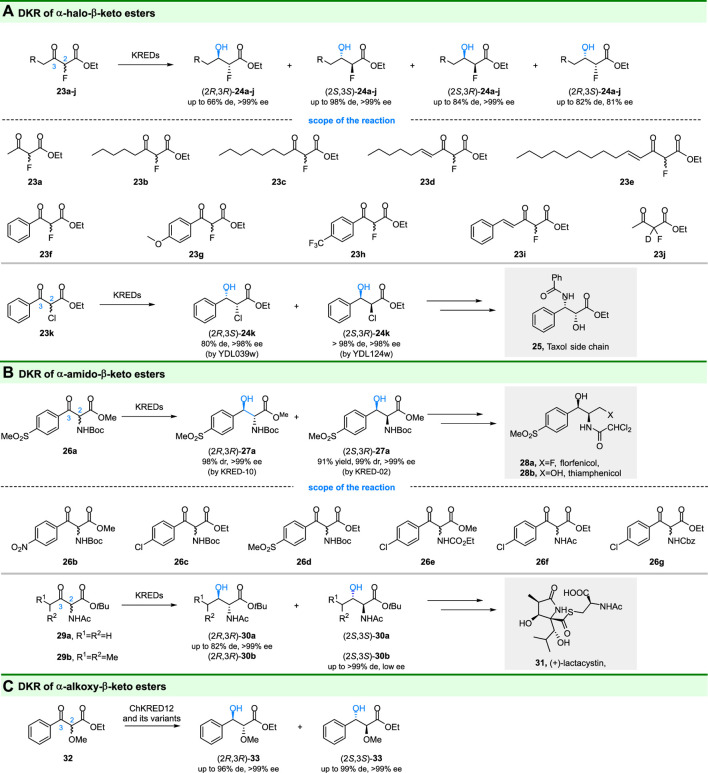
DKR of α-heteroatom substituted-β-keto esters. **(A)** DKR of α-halo-β-keto esters, **(B)** DKR of α-amido-β-keto esters, and **(C)** DKR of α-alkoxy-β-keto esters.

The C-F bond of α-fluoro-β-keto esters can affect the physical and chemical properties of the compound tremendously (O'Hagan, 2008), and fluorine also has a great influence on the biological activity of pharmacologically active compounds and drugs (Meanwell, 2018). Accordingly, Green and coworkers focus on selective catalytic reduction of α-fluoro-β-keto esters (**23a-i**) by DKR (Green et al., 2019) (Figure 6A). Using commercially available ketoreductases, most of the substrates could be reduced to corresponding α-fluoro-β-hydroxy esters in high optical purities and yields. Importantly, either anti- or syn-diastereomers could be produced with high diastereomeric and enantiomeric excess (Figure 6A), depending on the ketoreductases used. The authors also utilized in situ ^19^F NMR spectroscopy to monitor the enzyme reaction in real-time, which is convenient for analyses. The DKR of α-chloro-β-keto ester (**23k**) was also reported by Stewart and coworkers (Kaluzna et al., 2005b). In their study, syn-(2*R*,3*S*)-**24k** and syn-(2*S*,3*R*)-**24k** were obtained by recombinant reductases from *Saccharomyces cerevisiae*, and a chemoenzymatic route for synthesis of the side chain of Taxol (25) was developed. In addition, Berkowitz and coworkers also succeeded in gaining optical pure (2*S*,3*R*)-**24k** (95% de, 99% ee) by DKR of **23k** using an alcohol dehydrogenase from *Clostridium acetobutylicum* (*Ca*ADH) (Applegate et al., 2011).

Chiral 1,2-amino alcohols are important building blocks in fine chemistry and pharmaceutical synthesis. Ketoreductases catalyzed DKR of α-amido-β-keto esters is an attractive strategy to obtain optical pure 1,2-amino alcohols (Figure 6B). For example, Chen, Zhang and coworkers reported a ketoreductases based DKR process, in which, *cis*-(2*S*,3*R*)-**27a** with 99% dr and >99% ee was obtained by KRED-02 while *trans*-(2*R*,3*R*)-**27a** with 98% dr and >99% ee was obtained by KRED-10 (Zou et al., 2018). Importantly, a chemoenzymatic route for the synthesis of the broad-spectrum antibiotic florfenicol was developed based on the obtained *cis*-(2*S*,3*R*)-**27a**. Their work showed that the ketoreductases catalyzed DKR has industrial opportunities for applications. Using the same strategy, Chen and colleagues prepared a series of 1,2-amino alcohols [(2*S*,3*R*)-**26b-g**] through reduction of α-amido-β-keto esters by another ketoreductase WTEA (from *Exiguobacterium* sp. F42) and its evolved variants (Tang et al., 2021).

The other α-amido-β-keto esters (**29a-b**) have also drawn great attention since they are important building blocks for some natural products and pharmaceutical [e.g., ( + )-lactacystin (**31**)] (Seashore-Ludlow et al., 2012). Smonou and coworkers developed a DKR approach for reduction of **29a-b**, in which KRED-101 (Codexis) catalyzed reduction gave anti-**30a-b** with up to >99% conversion and >99% de values (Figure 5B) (Giannopoulos et al., 2020). However, this enzyme displayed poor enantioselectivity [for (2*S*,3*S*)-**30b**], hence, it was partially successful in the synthesis of lactacystin’s precursor. Nevertheless, in the various screened ketoreductases, KRED-119 showed moderate activity (40% conversion) and gave undesired (2*R*,3*R*)-**30a** with 82% de and >99% ee values.

The biocatalytic reduction of racemic α-alkoxy-β-keto esters *via* DKR to prepare 1,2-diols was first reported by Li C. et al. (2020). The structure of chiral 1,2-diols consists widely of natural products and also serves as building blocks for several useful compounds. Racemic ethyl 2-methoxy-3-oxo-3-phenylpropanoate (**32**) could be stereoselectively reduced to (2*S*,3*S*)-**33** with >99% ee and >99:1 dr by *Ch*KRED12 M191S variant (Figure 6C). The *Ch*KRED12 Q151Y/M191L variant displayed reversed stereoselectivity and gave (2*R*,3*R*)-**33** with >99% ee and 98:2 dr. Although the other diastereoisomers were not obtained, the DKR can probably explore the substrate spectrum of the *Ch*KRED12 and its mutants.

### 3.2 Ketoreductase Involved in DKR of α-Alkyl-Substituted β-Keto Esters

The α-alkyl-β-hydroxy esters are important building blocks in organic synthesis and pharmaceutical synthesis. Ketoreductases catalyzed DKR of α-alkyl-β-keto esters is an important strategy to prepare optical pure α-alkyl-β-hydroxy esters. Using the DKR method, Kambourakis, Smonou and coworkers tested several α-alkyl substituted-β-keto esters by ketoreductases and microbial reductions. For example, KRED-108 displayed high (2*R*,3*R*)-selectivity towards **34d** and **34f**, yielding corresponding products with >99% de and >99% ee (Kambourakis and Rozzell, 2003; Kambourakis and Rozzell, 2004). Meanwhile, another enzyme KRED 102 could give optical pure (>99% de and >99% ee) (2*R*,3*S*)-**35g** and (2*R*,3*S*)-**35h** by reduction of **34g** and **34h**, respectively (Kalaitzakis et al., 2005). Gotor and colleagues also reported that the “small-bulky” prelog ADHs could reduce a series of α-alkyl-β-keto esters (**34g**, **34l-t**) to the corresponding (2*R*,3*S*)-alcohols with >99% ee and 90–99% de values (Cuetos et al., 2012).

**FIGURE 7:**
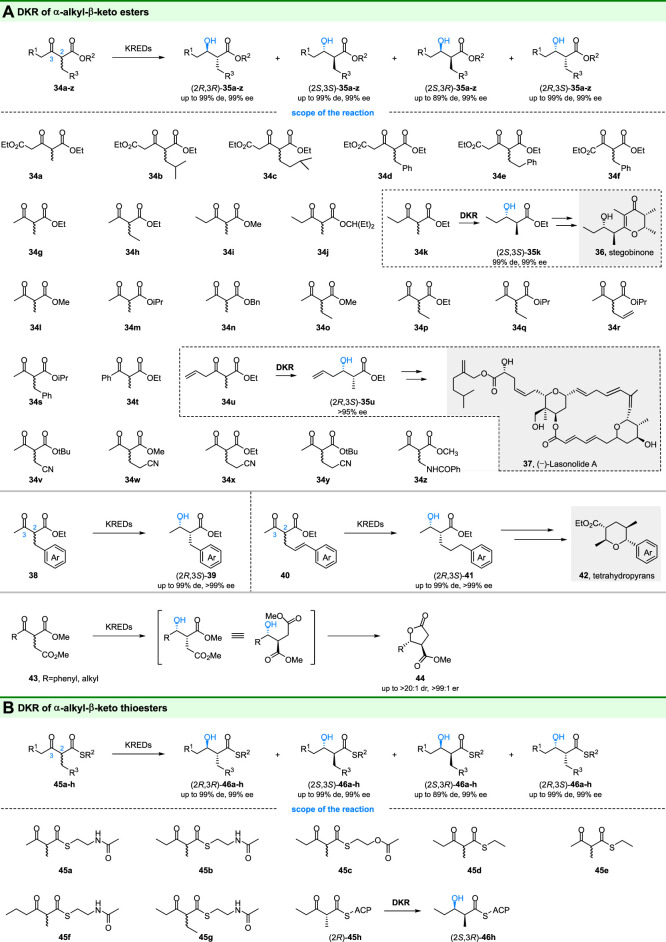
DKR of **(A)** α-alkyl-β-keto esters and **(B)** α-alkyl-β-thioesters esters.

Importantly, the obtained optical pure α-alkyl-β-hydroxy esters are useful for synthesis of pharmaceutically active compounds. As shown in Figure 7A, the (2*S*,3*S*)-**35k** is a precursor for the synthesis of stegobinone (**36**), a natural sex pheromone of the drugstore beetle (Burkholder and Ma, 1985; Kalaitzakis and Smonou, 2012). Thus, a chemoenzymatic route for the synthesis of stegobinone was a development based (2*S*,3*S*)-**35k** with 99% de and 99% ee from DKR of **34k**. Similarly, the (2*R*,3*S*)-**35u** from DKR of **34u** was used for the total synthesis of polyketide (−)-Lasonolide A (Trost et al., 2016). In addition, chiral α-cyanomethyl-β-hydroxy esters, which are chiral intermediates for the synthesis of α-substituted γ-butyrolactams, could also be obtained by ketoreductases catalyzed DKR of α-cyanomethyl-β-ketones esters (**34v-y**) (Giannopoulos et al., 2020). Zheng and co-workers also achieved the stereoselective DKR of α-benzamido-β-ketoester (**34z**) by using different recombinant enzymes and gave (2*S*,3*R*)-**35z** with 99% de and 99% ee, which is a significant building block for carbapenems (Chen et al., 2015). These DKR reactions amplified the applications of ketoreductases in environmentally friendly processes.

Another study by Nanda group reported that the ketoreductase from *Klebsiella pneumoniae* could convert α-benzyl/cinnamyl substituted-β-ketoesters (**38** and **40**) to the α-benzyl/cinnamyl substituted-β-hydroxy esters through DKR (Barik et al., 2019) (Figure 7A). Not only mono-aryl rings but also naphthyl/heteroaryl rings can be accepted by the ketoreductase with excellent ee (99%) and de (99%). For α-cinnamyl substituted-β-hydroxy esters, they can be further synthesized into substituted tetrahydropyran derivatives (**42** in Figure 7A), which are present in some natural products.

The γ-lactones are usually found in natural products and methods for selective construction of these compounds are highly desired. Recently, Scheidt and coworkers developed a chemoenzymatic strategy for the synthesis of γ-lactones based on enzymatic DKR and intramolecular cyclization (Maskeri et al., 2020). Commercially available KRED-P2-C02 (Codexis) was chosen to catalyze the reduction of substrates (**43**) and gave the γ-lactones (**44**) with high enantio- and diastereoselectivity. They also established that the optimal solvent condition for catalytic reduction was 1:3:6 isopropanol:1-chlorododecane:0.1 M tris buffer at pH = 9.

As is well-known, ketoreductase domains (KR domains) were involved in modular polyketide synthases (PKS). Ludeke and coworkers demonstrated that Tyl-KR1, an isolated PKS ketoreductase domain in the biosynthesis of tylactone, has reductive activity toward α-alkyl-β-keto thioesters **45a** and **45b** (Figure 7B), but with medium selectivity (Häckh et al., 2013). In addition, through structure-activity relationship study and rational engineering, the Keatinge-Clay group investigated the stereocontrols of ketoreductase domains of modular PKS. The reverse stereoselectivity of EryKR1, from (2*S*,3*R*)- to (2*S*,3*S*)-, was found in the reduction of **45b-d** by mutations (Bailey et al., 2016). Bailey and coworkers also reported the engineering of modular PKS ketoreductase domains for altering their stereochemical controls (reduction of **45h** for an example) (Drufva et al., 2020).

### 3.3 Ketoreductase Involved in DKR of α-Alkyl-β-Keto Amides and α-Substituted β-Arylphosphonates

**FIGURE 8:**
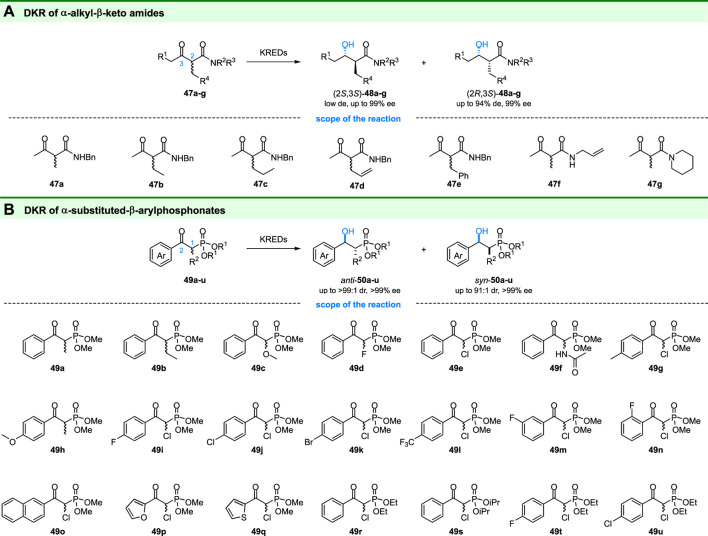
DKR of **(A)** α-alkyl-β-keto amides and **(B)** α-substituted-β-arylphosphonates.

Chiral α-substituted-β-hydroxy amides are important building blocks of some biologically active compounds (Zhang et al., 2020). The stereoselectivity synthesis of these compounds is still a challenge although various synthetic strategies have been developed. Lavandera and coworkers reported that these compounds can be acquired by ADHs catalyzed DKR of α-alkyl-β-ketone amides (Méndez-Sánchez et al., 2019) (**47a-g** in Figure 8A). Through screening a set of ADHs, syn-α-alkyl-β-hydroxy amides could be obtained with high diastereo- and enantioselectivities (Figure 8A). In addition, the ADH-A from *Rhodococcus ruber* can get access to the diastereoisomers (2*R*,3*S*)-**48a-f** with 59–95% de and >99% ee at 25 mM scales.

Recently, Chen and coworkers reported the ketoreductases catalyzed DKR of α-substituted β-keto arylphosphonates (Wang et al., 2020) (**49a-u** in Figure 8B). 14 KREDs were tested for their reductive ability on diverse α-substituted β-keto arylphosphonates. And most of these substrates could be reduced to corresponding α-substituted-β-hydroxy arylphosphonateshonates (**50a-u** in Figure 8B) in high isolated yield (up to 96%). Depending on the ketoreductases used, anti- or syn-products could be obtained with good-to-excellent diastereomeric purity (up to >99:1 dr) and excellent enantiomeric purity (up to >99% ee). This is the first systematic study of KRED-catalyzed DKR of β-keto arylphosphonates. Interestingly, the obtained anti-(1*R*,2*R*)-**49u** exhibited weak antibacterial activities.

### 3.4 Ketoreductase Involved in DKR of α-Substituted Ketones

**FIGURE 9:**
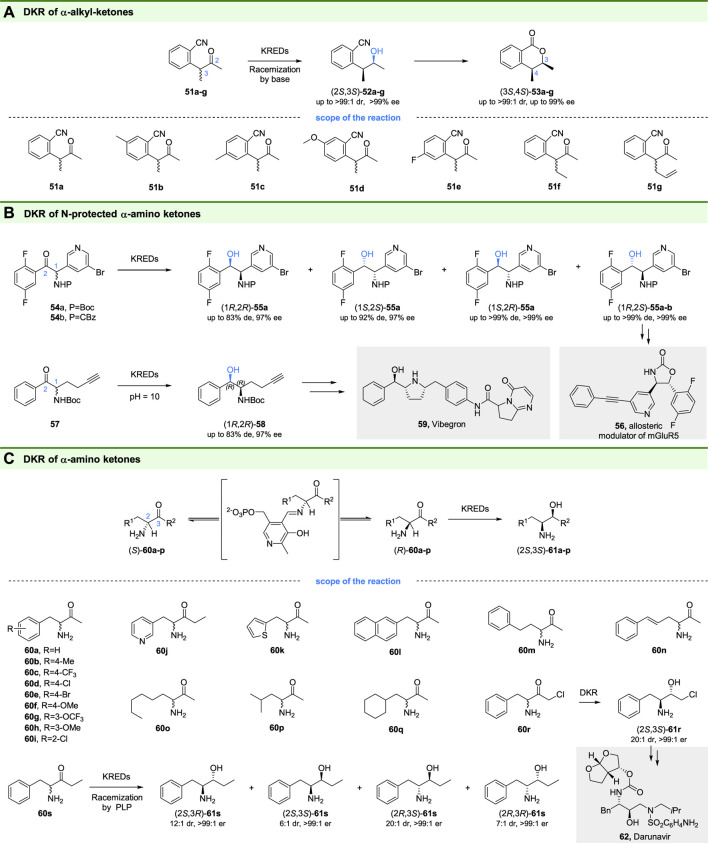
DKR of α-substituted ketones. **(A)** DKR of α-alkyl ketones, **(B)** DKR of N-protected α-amido ketones, and **(C)** DKR of α-amido ketones.

Chiral heterocyclic compounds and their derivatives, such as 3,4-dihydroisocoumarins, are ubiquitous in natural products and usually have biological activities of antifungal, antimalarial, anticancer, and antibacterial (Jiao et al., 2006; Sharma et al., 2010). Therefore, the development of synthetic methods for these compounds is highly desired. In 2013, Gotor-Fernández and coworkers reported a chemoenzymatic route for the synthesis of enantiopure 4-alkyl-3-methyl-3,4-dihydroisocoumarins based ketoreductases catalyzed DKR (Mangas-Sánchez et al., 2013). They found that the ADH-A from *Rhodococcus ruber* exhibited excellent diastereo- and enantioselectivity toward 2-(3-oxobutan-2-yl) benzonitriles (**51a-g** in Figure 9A) and gave the (2*S*,3*S*)-4-alkyl-3-methyl-3,4-dihydroisocoumarins (**52a-g** in Figure 9A) as the products. By using anion exchange resin DOWEX MWA-1 as an additive for racemization, a DKR process for the reduction of racemic ketones was constructed (Figure 9A). The obtained enantiopure alcohols could be subsequently cyclized to corresponding lactones (3*S*,4*S*)-**53a-g** in one pot with good to excellent conversions (72–96%) and high selectivity (up to >99:1 dr and >99% ee).

Vicinal amino alcohols are useful building blocks in the synthesis of natural products and pharmaceuticals (Orcel and Waser, 2015). For example, the (1*R*,2*S*)-**55a** is an important intermediate for the synthesis of compound **56**, a mGluR5 allosteric modulator developed for the treatment of neurological disorders. DKR of N-protected α-amino ketones is an interesting strategy for preparation of the chiral amino alcohols, however, only a few studies on DKR of N-protected α-amino β-keto esters have been reported (Zou et al., 2018; Giannopoulos et al., 2020). To prepare optically pure (1*R*,2*R*)-**55a,** the scientists at Bristol-Myers Squibb recently developed a DKR process of N-protected α-amino ketones (Hanson et al., 2016). Through screening, hundreds of commercial ketoreductases and microbial strains, the desired syn-(1*R*,2*S*)-**55a** and the other three diastereoisomers could be obtained with excellent enantiomeric excess and diastereoisomeric excess through DKR. The obtained chiral alcohols (1*R*,2*S*)-**55a-b** with >99% de and >99% ee by KRED-145 catalyzed DKR has been used for the synthesis of the drug candidate **56** (González-Bobes et al., 2016).

Similarly, the scientist at Merck and Codexis also reported the DKR of N-protected α-amino ketone **57** (Xu et al., 2018). Unlike the compounds **54a-b,** the racemization of which occurs at pH 7, the fast racemization of **57** could only be realized at elevated temperature (≥45°C) and high pH (≥ pH 10), while these conditions were challenges for ketoreductases. Thus, through the incorporation of structurally guided design and directed enzyme evolution, a ketoreductase suitable for high-pH and elevated-temperature conditions was developed. The DKR of **57** by evolved KRED-p301 gave the desired (1*R*,2*R*)-**58** with >99% ee and >100:1 dr. Finally, a chemoenzymatic route for the synthesis of Vibegron (**59**), an approved drug for the treatment of overactive bladder, was developed based (1*R*,2*R*)-**58** from DKR.

Although successful DKR of N-protected α-amino ketones has been reported, the DKR of unprotected α-amino ketones seems more attractive since no deprotection is needed. However, substrates for ketoreductases catalyzed DKR require acidic α-proton sites with pKa’s between 7 and 12, while the most α-amino ketones have less acidic α-protons, indicating the limitation of racemization under mild conditions. To overcome this limitation, Hyster and coworkers introduced pyridoxal-5-phosphate (PLP) into DKR as a catalyst to racemize starting racemic α-amino ketones (Cao and Hyster, 2020). By using commercial KRED P2-D11 and P1-B05, the DKR of α-amino ketones (**60a-p**) gave the 1,2-amino alcohols (2*S*,3*S*)-**61a-q** with high yields (85–99% yield) and high selectivities (up to >20:1 dr, >99:1 er). In addition, by using KRED P1-B10, another diastereoisomer (2*S*,3*S*)-**61r** could be obtained, which could be used for the synthesis of antiviral drugs Darunavir (**62**) and Atazanavir. Moreover, stereodivergent synthesis of all four diastereoisomers of **61s** could also be achieved through DKR of **60s** using different ketoreductases, highlighting the benefit of PLP and KRED based DKR.

### 3.5 Ketoreductase Involved in DKR of Cyclic Ketones

**FIGURE 10:**
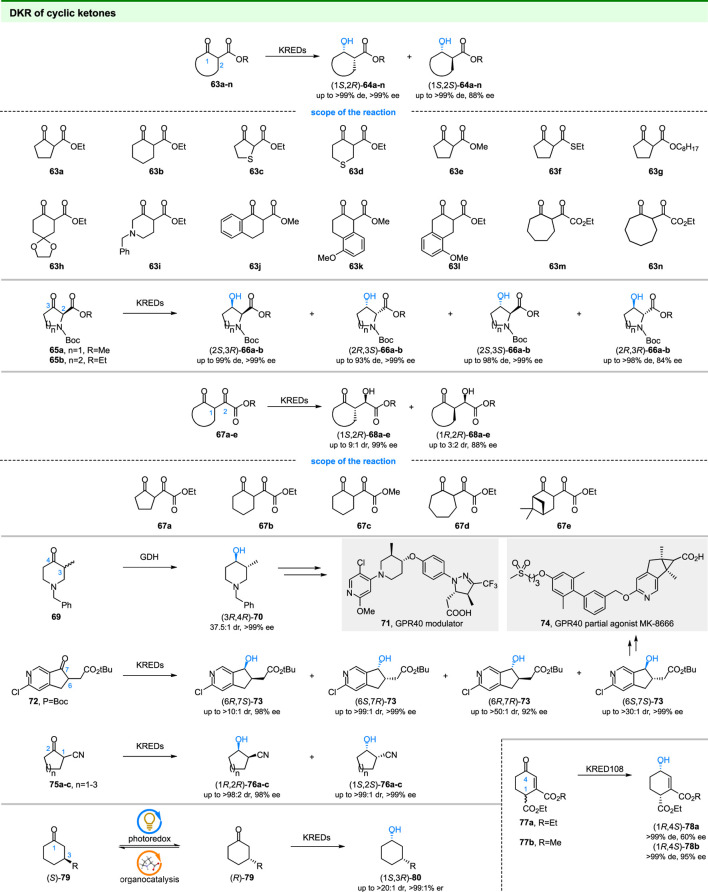
DKR of cyclic ketones.

Microbial reduction of cyclic β-keto esters (**63a-n** in Figure 10) has been well studied in last century. In early efforts of microbial reductions, baker’s yeast usually served as the catalyst and showed high selectivity in some cases. For example, the reduction of **63a-b** and **63h** by ‘non-fermenting’ baker’s yeast gave the (1*S*,2*R*)-products with >99% de and 99% ee values (Seebach et al., 1987). In addition, the baker’s yeast has (1*S*,2*R*)-diastereopreference and displayed >98% diastereoselectivity in the reduction of **63f-g** and **63j-l** (Seebach et al., 1987; Sato et al., 1988). (1*S*,2*S*)-products could also be obtained. For example, reduction of **63m** and **63n** by *Mucor griseocyanus* gave corresponding (1*S*,2*S*)-**63m** and (1*S*,2*S*)-**63n** with 76% de and >99% de values (Danchet et al., 1997). DKR of cyclic β-keto esters have also been investigated by using isolated ketoreductases. For example, the scientists at Merck achieved the stereodivergent synthesis of 3-hydroxyproline (**66a**) and 3-hydroxypipecolic acids (**66b**) by DKR of **65a-b** using commercial ketoreductases (Prier et al., 2019).

Similar to cyclic β-keto esters, the DKR of cyclic α, γ-diketo esters (**67a-e** in Figure 10) has also been reported by using baker’s yeast (Tsuboi et al., 1987). The results indicated that the reduction of **67a-e** with fermenting baker’s yeast afforded chiral 2-hydroxy-2-(2-oxocycloalkane)acetates, which were useful for the synthesis of natural products. Interestingly, the bakers’ yeast has (1*S*,2*R*)-diastereopreference toward **67b-e** and gave corresponding products with 3:2 to 9:1 dr. Similar to the cyclic α, γ-diketo esters, the DKR of linear β, δ-diketo esters could also be achieved by KREDs (Wolberg et al., 2001; Lüdeke et al., 2009).

The chiral cyclic alcohols are also building blocks in the synthesis of pharmaceuticals. In 2016, the scientists at Bristol-Myers Squibb and Merck reported the synthesis of GPR40 modulator (**71**) (Hanson et al., 2016) and GPR40 partial agonist MK-8666 (**74**) (Hyde et al., 2016) using chiral cyclic alcohols obtained from DKR of **69** and **72**, respectively (Figure 10). In the first study, a series of ketoreductases were screened for DKR of **69**. Surprisingly, the glucose dehydrogenase (GDH) displayed the highest selectivity among the tested enzymes and gave the desired (3*R*,4*R*)-**70** with 37.5:1 dr and >99% ee (Hanson et al., 2016). In the next study, the scientists at Merck screened several commercial KREDs to produce **73**, a key intermediate for MK-8666, by DKR of **72** (Hyde et al., 2016). Stereodivergent synthesis of all four diastereoisomers of **73** was achieved by KREDs with different stereo preferences and the desired (6*S*,7*S*)-**73** was obtained with >30:1 dr and >99% ee by optimized KRED 264. And a chemoenzymatic route for the synthesis of MK-8666 was developed based on DKR.

At the same time, the couple of ketoreductases catalyzed DKR and nitrile hydratases/amidases catalyzed hydrolysis was reported to provide enantiopure β-hydroxy carboxylic acids by Rebolledo and coworkers (Liardo et al., 2016). For the first step, the authors utilized commercially available KREDs to reduce 2-oxocycloalkanecarbonitriles (**75a-c** in Figure 10). The diastereoisomers (1*R*,2*R*)-**76a-c** and (1*S*,2*S*)-**76a-c** could be obtained with high selectivity (88:12 to >99:1 dr and 95% to >99% ee) by using different KREDs. For the next step, well-studied *Rhodococcus rhodochrous* were used for hydrolysis of 2-oxocycloalkanecarbonitriles. Finally, the bio-cascade process gave the β-hydroxy carboxylic acids with excellent overall yield and optical purity.

As shown in Figure 10, another type of substrate for DKR was α, β-unsaturated cyclic ketones (Kosjek et al., 2006). Transition metal-based hydrogenation of **77a-b** gave the allylic alcohol in low diastereomeric excess. In contrast, the enzymatic reduction of **77a-b** resulted in both high enantio- and diastereoselectivity. The obtained chiral allylic alcohols are important building blocks in the synthesis of natural compounds.

In contrast to the aforementioned and well-studied DKR of α-substituted ketones, DKR of β-substituted ketones is still challenging since the lack of racemization methods for static β-keto stereocenters under mild conditions. In 2020, the Macmillan and Hyster groups describe a general method for the racemization of traditionally static β-keto stereocenters by a combination of photoredox catalysis and organocatalysis (DeHovitz et al., 2020). The method was then subjected to ketoreductases catalyzed reduction to achieve the DKR of β-substituted ketones **79**. Related alcohols (1*S*,3*R*)-**80** could be obtained with high yields (68%–82%) and high selectivities (up to >20:1 dr, >99:1 er) by a combination of photoredox- and enzyme-catalysis. The stereodivergent synthesis of all four stereoisomers of **80** could also be achieved by using ketoreductases with different stereopreferences. In addition, other enzymes, such as transaminases, could be incorporated into this DKR process instead of ketoreductases, indicating the power of this strategy toward static stereocenters.

## 4 Discussion and Conclusion

Biocatalyzed (dynamic) kinetic resolution is an environmentally friendly strategy for the preparation of chiral compounds. In this review, we summarized a series of examples of KREDs catalyzed kinetic resolution (KR) or dynamic kinetic resolution (DKR). With the development of enzyme mining and protein engineering, KREDs have been successfully applied to KR or DKR of several ketone compounds, including but not limited to α-substituted β-keto esters, α-alkyl-β-keto amides, α-substituted β-arylphosphonates, α-substituted β-ketones, and cyclic ketones. All the summarized KR or DKR reactions could produce corresponding chiral products with at least two chiral centers. In addition, the KRED-based KR and DKR also show potential for industrial applications. Despite this, challenges or limitations still remain, especially in DKR. As mentioned, the KREDs catalyzed DKR relies on efficient racemization between two enantiomers and selective reduction of one of them by KREDs. First, racemization in most reviewed DKR is spontaneous, a general racemization method for substrates that cannot be racemized spontaneously is lacking. Although Macmillan and Hyster groups showed a remarkable example for racemization of static stereocenters (DeHovitz et al., 2020), other studies are rarely reported, especially in comparison with the successful application of metal catalysts for racemization of secondary alcohols in lipase-mediated DKR (Zhu, 2021). Second, as reviewed, a lot of screening efforts were performed to get the desired alcohols in KRED catalyzed reduction reactions. However, lower yields or wrong stereoisomers were obtained in some cases. Thus, the ketoreductases with higher selectivity, activity, stability, and tolerance to adverse reaction conditions are still needed. Nevertheless, the increasing studies on protein engineering or directed evolution of KREDs, and combining KREDs with other catalysts, such as biocatalysts, metal catalysts (Burda et al., 2008; Ríos-Lombardía et al., 2016), organocatalysts (Baer et al., 2009; Rulli et al., 2011; Rudroff et al., 2018), photocatalysts (DeHovitz et al., 2020), and electrocatalysts (Wan et al., 2021), in one-pot or by consecutive reactions, further encourage the application of KREDs in asymmetric synthesis and also (dynamic) kinetic resolution. We finally believe that the novel methods of racemization, the further engineering of KREDs, and the combination of KREDs with other catalytic systems will continue to encourage the applications of KREDs catalyzed (dynamic) kinetic resolutions for biomanufacturing of chiral chemicals.
